# Clinical significance and potential molecular mechanism of miRNA-222-3p in metastatic prostate cancer

**DOI:** 10.1080/21655979.2020.1867405

**Published:** 2021-01-12

**Authors:** Yu Sun, Gang Chen, Juan He, Zhi-Guang Huang, Sheng-Hua Li, Yuan-Ping Yang, Lu-Yang Zhong, Shu-Fan Ji, Ying Huang, Xin-Hua Chen, Mao-Lin He, Hao Wu

**Affiliations:** a Division of Spinal Surgery, The First Affiliated Hospital of Guangxi Medical University, Nanning, P.R. China; b Department of Pathology, The First Affiliated Hospital of Guangxi Medical University, Nanning, P.R. China; c Department of Urology, The First Affiliated Hospital of Guangxi Medical University, Nanning, P.R. China

**Keywords:** miRNA-222-3p, metastasis, prostate cancer, synaptosome associated protein 91, biomarker

## Abstract

The clinical significance and underlying molecular mechanism of miRNA-222-3p in metastatic prostate cancer (MPCa) remain unclear. The present study used a large number of cases (n = 1,502) based on miRNA chip and miRNA sequencing datasets to evaluate the expression and diagnostic potential of miRNA-222-3p in MPCa. We applied a variety of meta-analytic methods, including forest maps, sensitivity analysis, subgroup analysis and summary receiver operating characteristic curves, to prove the final results. MiRNA-222-3p was reduced in MPCa and had a moderate diagnostic potential in MPCa. We screened 118 miRNA-222-3p targets using three different methods including miRNA-222-3p transfected MPCa cell lines, online prediction databases and differently upregulated genes in MPCa. Moreover, functional enrichment analysis performed to explore the potential molecular mechanism of miRNA-222-3p showed that the potential target genes of miRNA-222-3p were significantly enriched in the p53 signal pathway. In the protein–protein interaction network analysis, SNAP91 was identified as a hub gene that may be closely related to MPCa. Gene chip and RNA sequencing datasets containing 1,237 samples were used to determine the expression level and diagnostic potential of SNAP91 in MPCa. SNAP91 was found to be overexpressed in MPCa and had a moderate diagnostic potential in MPCa. In addition, miRNA-222-3p expression was negatively correlated with SNAP91 expression in MPCa (r = −0.636, P = 0.006). These results demonstrated that miRNA-222-3p might play an important role in MPCa by negatively regulating SNAP91 expression. Thus, miRNA-222-3p might be a potential biomarker and therapeutic target of MPCa.

## Graphical abstract

**Figure:**
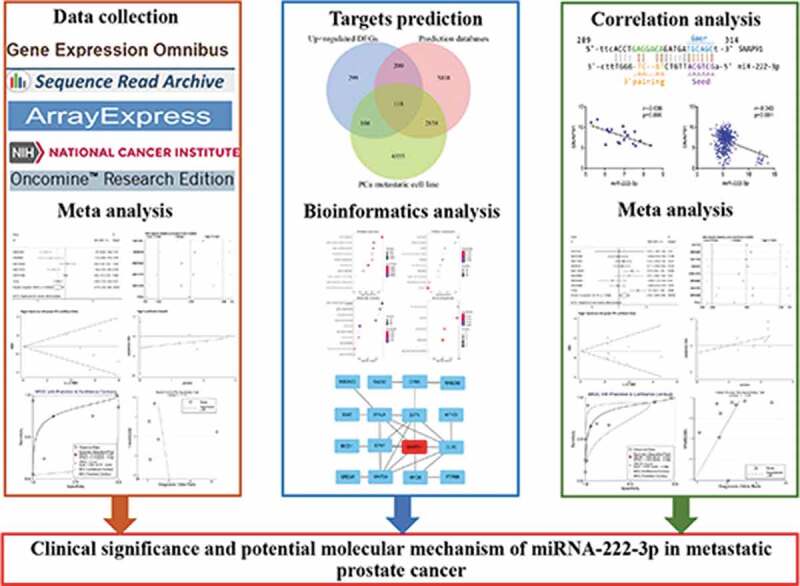

## 1. Introduction

Prostate cancer (PCa) is caused by malignant hyperplasia of prostate epithelial cells. It is one of the most common cancers of the male genitourinary system worldwide and has a high incidence among male malignant tumors [1–3]. In patients with localized prostate cancer (LPCa), radical prostatectomy, radiotherapy and hormone deprivation therapy can markedly beneficial effects, prolonging the survival time of patients [4–10]. However, due to the lack of obvious symptoms and specific biomarkers in the early stage of PCa metastasis, the diagnosis and treatment of metastatic prostate cancer (MPCa) is commonly delayed. Once PCa metastasis has occurred, the options for treatment are limited, leading to a reduction in survival, a decline in the patient’s quality of life, and eventually to death. Therefore, elucidating the molecular mechanism of PCa metastasis is very important for the identification of novel strategies for the diagnosis and treatment of MPCa.

MicroRNAs (miRNAs) are short, non-coding, endogenous RNAs that participate in a variety of biological processes by negatively regulating the expression of target genes [11–16]. Recent studies have shown that miRNAs were involved in MPCa. For example, miRNA-92a has been shown to be downregulated in PCa cells and inhibit PCa cell viability and metastasis by targeting SOX4 [17]. MiRNA-1236-3p inhibited the growth and metastasis of PCa cells by inhibiting the TLR2 and AKT pathways, and activating p21 [18]. The upregulated expression of the long non-coding RNA PVT1 induced PCa metastasis by targeting miRNAs (miRNA-15b-5p, miRNA-27a-3p, miRNA-143-3p and miRNA-627-5p) [19]. MiRNA-222-3p has been shown to play an important role in the metastasis of various tumors. Peng et al. reported that miRNA-222-3p promoted ovarian cancer metastasis by regulating HOXC10 [20]. MiRNA-222-3p has also been shown to be highly expressed in patients with thyroid papillary carcinoma metastasis, and its upregulation enhanced the metastatic and invasiveness ability of cancer cells [21]. The expression of miRNA-222-3p was increased in clinical specimens and cell lines of renal cell carcinoma and correlated positively with the metastatic potential of renal cell carcinoma [22]. MiRNA-222-3p was highly expressed in non-small cell lung cancer and mediated metastasis by activating the SOCS3/Stat3 signal pathway [23]. In contrast, miRNA-222-3p was significantly downregulated in epithelial ovarian cancer and inhibited cell metastasis in vitro [24]. However, contradictory results were obtained in the only two reports of the expression of miRNA-222-3p in MPCa, which were conducted with a small sample size [15,25]. In addition, the clinical significance and potential molecular mechanism of miRNA-222-3p in MPCa are still unclear.

In this study, we obtained the expression data of miRNA-222-3p in MPCa from miRNA chip and miRNA sequencing datasets and calculated the standard mean difference and summary receiver operating characteristic to evaluate the expression level and diagnostic potential of miRNA-222-3p in MPCa. The potential molecular mechanism of miRNA-222-3p in MPCa was explored through functional enrichment analysis. SNAP91 had the highest degree of connectivity in the protein–protein interaction (PPI) network analysis and was identified as a hub gene of miRNA-222-3p. Thus, we speculated that miRNA-222-3p participates in the metastasis of PCa by negatively regulating the expression of SNAP91, which was verified by prediction tools and correlation analysis.

## 2. Materials and methods

### 2.1. Collection of miRNA-222-3p expression data in MPCa and PCa

To obtain miRNA-222-3p expression datasets from the Gene Expression Omnibus (GEO), ArrayExpress, Sequence Read Achieve (SRA), The Cancer Genome Atlas (TCGA) and Oncomine databases, the following search terms were applied: (parastata OR prostatic gland OR prostate gland OR prostat*) AND (cancer OR carcinoma OR tumor OR neoplas* OR malignan* OR adenocarcinoma) AND (miR OR miRNA OR microRNA). The retrieval results were in accordance with the following inclusion criteria: (1) studies involving two groups (MPCa vs. LPCa or PCa vs. non-PCa), (2) the studies were conducted in humans, and (3) the sample size of MPCa and LPCa was ≥3. Studies without miRNA-222-3p expression data were considered ineligible and therefore excluded.

### 2.2. Screening potential miRNA-222-3p target genes in MPCa

#### 2.2.1. MPCa cell lines transfected with miRNA-222-3p

Gene expression profiles of MPCa cell lines transfected with miRNA-222-3p analogs or inhibitors were acquired from the GEO database. The potential target genes of miRNA-222-3p were selected from those identified in MPCa cell lines with changes in expression filtered according to the following criterion: (|log2FC| > 1).

#### 2.2.2. Upregulated differentially expressed genes (DEGs) in MPCa

Ten studies of MPCa were screened based on the databases and search formulas noted in Section 2.1. Generally speaking, miRNAs exert its biological function by negatively regulating the expression of target genes. In this study, our results showed that miRNA-222-3p was downregulated in MPCa. Therefore, the upregulated DEGs in MPCa were more likely to be potential target genes of miRNA-222-3p. The upregulated DEGs in the mRNA sequencing datasets and gene chip results were retrieved using the Limma-Voom package and the Limma package, respectively, with |log_2_FC| > 1 and adjusted P < 0.05 as the filtering criteria. Genes that appeared at least three times among the groups of upregulated DEGs were deemed to be the potential target genes of miRNA-222-3p.

#### 2.2.3. The online prediction database

The target genes of miRNA-222-3p were predicted by miRWalk2.0, which is an online prediction database consisting of 12 miRNA-mRNA prediction platforms. The target genes of miRNA-222-3p were predicted by at least five platforms were selected for further analysis. The potential target genes of miRNA-222-3p were determined by the overlap of the outcomes from the three sources described.

### 2.3. GO, KEGG and PPI network analyses

GO function and KEGG analyses were conducted utilizing DAVID6.8 to identify the potential molecular mechanisms of candidate target genes of miRNA-222-3p. The PPI network was visualized using the STRING database and Cytoscape software. SNAP91 showed the highest connectivity among the hub genes and was selected for subsequent analysis.

### 2.4. Correlation analysis of miRNA-222-3p and SNAP91

Datasets including both miRNA-222-3p and SNAP91 expression levels in MPCa and LPCa were acquired from TCGA database. The association between miRNA-222-3p and SNAP91 was evaluated using Spearman’s correlation coefficient test. In addition, the correlations between miRNA-222-3p and SNAP91 in different cancers were assessed using StarBase v3.0.

### 2.5. The clinical significance of SNAP91 in MPCa

Based on the databases and the filtering strategy used in this study, we retrieved the SNAP91 mRNA profiles in MPCa and PCa. The Human Protein Atlas (HPA) was used to verify SNAP91 protein expression in PCa.

### 2.6. Statistical analysis

The miRNA-222-3p and SNAP91 expression data were normalized and log2-transformed. Statistical analysis was performed with SPSS22.0 software (SPSS, IBM, Chicago, IL, USA). Differences between two independent groups were compared using the independent-samples *t*-test and visualized by scatter plots, which were created with GraphPad Prism 5 (GraphPad Software, Inc., La Jolla, CA, USA). To determine the potential of miRNA-222-3p and SNAP91 in distinguishing the two independent groups, the summary receiver operating characteristic curves were generated and the area under the curve was calculated. To assess the trends in the expression of miRNA-222-3p and SNAP91 in two independent groups, the pooled standard mean difference with 95% confidence intervals (CI) was determined using Stata14.0 software (Stata Corporation, College Station, TX, USA). When P < 0.05 or I^2^ > 50%, a random‑effects model was selected; otherwise, the fixed‑effect model was used. Sensitivity and subgroup analyses were performed to explore the sources of the inter-study heterogeneity. Furthermore, Begg’s and Egger’s tests were used to assess potential publication bias. Kaplan–Meier survival curves were plotted and the log-rank tests were employed to assess patient survival rates. P < 0.05 was considered to indicate a statistically significant difference. A flow chart of the design of our study is shown in Supplemental Figure S1.

## 3. Results

In our study, we paid more attention to investigating the clinical significance and potential molecular mechanism of miRNA-222-3p in MPCa. We hope to find new biomarker and therapeutic targets for MPCa. We verified the clinical significance of miRNA-222-3p in MPCa by miRNA chip and miRNA sequencing datasets. The miRNA-222-3p targets were screened from miRNA-222-3p transfected MPCa cell lines, online prediction databases and differently upregulated genes in MPCa. Functional enrichment analysis was performed to explore the potential molecular mechanism of miRNA-222-3p. SNAP91 had the highest degree of connectivity in the PPI network analysis and was identified as a potential target gene of miRNA-222-3p. The relationship between miRNA-222-3p and SNAP91 was verified by prediction tools and correlation analysis. We speculated that miRNA-222-3p might be involved in the metastasis of PCa through negative regulation of SNAP91.

### 3.1. Clinical significance of miRNA-222-3p in MPCa

**Figure 1.**
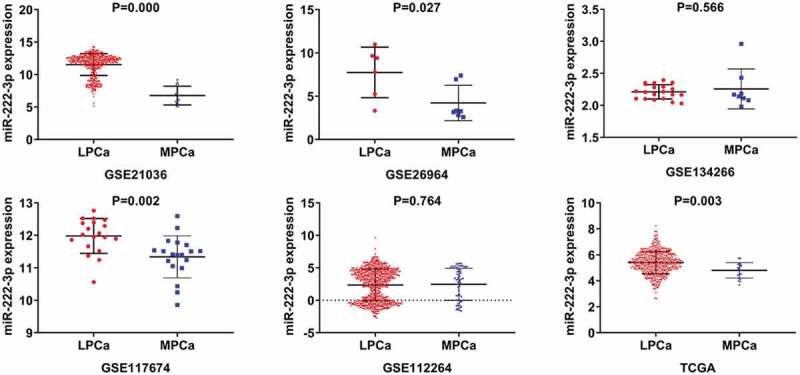
TCGA: The Cancer Genome Atlas; LPCa: localized prostate cancer; MPCa: metastatic prostate cancer

**Figure 2.**
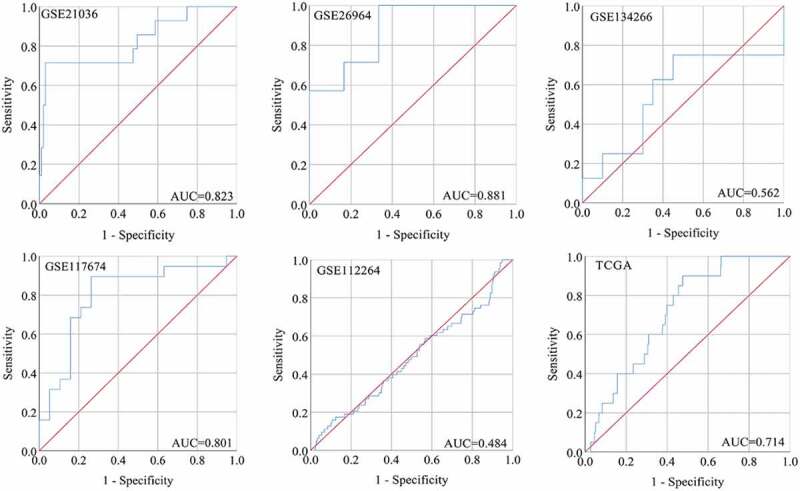
The ROC curves of miRNA-222-3p for distinguishing capacity in MPCa samples. area under the curve: area under the curve; TCGA: The Cancer Genome Atlas; ROC: receiving operator characteristic; MPCa: metastatic prostate cancer

**Figure 3.**
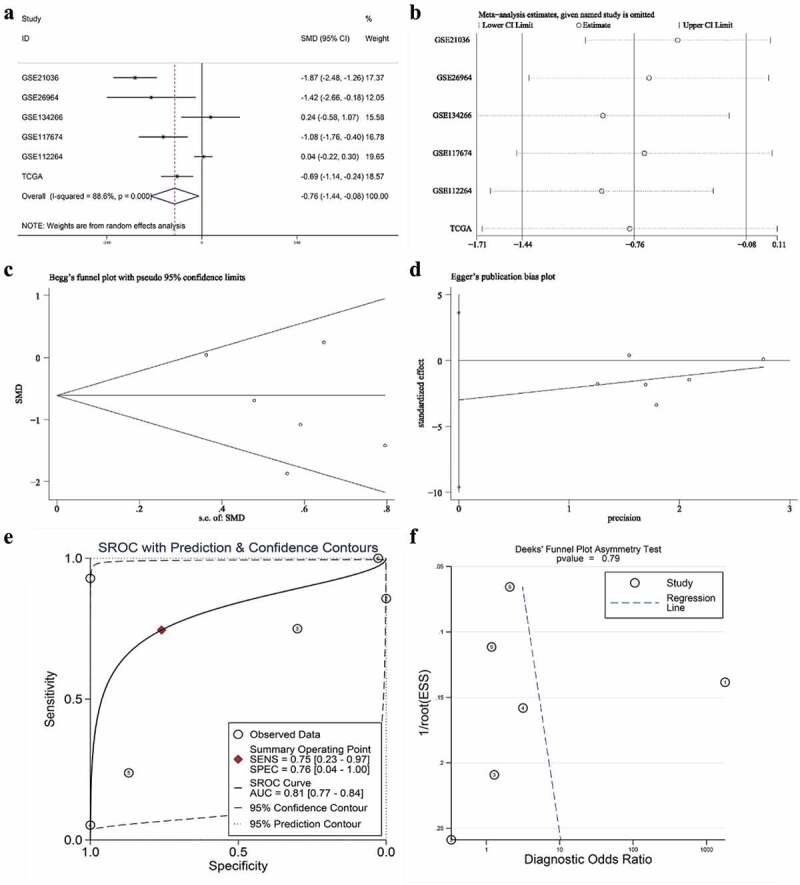
The expression and distinguishing capacity of miRNA-222-3p in MPCa. (a) Forest plot. (b) Sensitivity analysis plot. (c) The Begg’s funnel plot of the publication bias. (d) Egger’s publication bias plot. (e)SROC curve. (f) Deek’s funnel plot test. TCGA: The Cancer Genome Atlas; standard mean difference: standard mean difference; CI: confidence interval; area under the curve: area under the curve; SROC: summarized receiver operating characteristic; MPCa: metastatic prostate cancer

**Figure 4.**
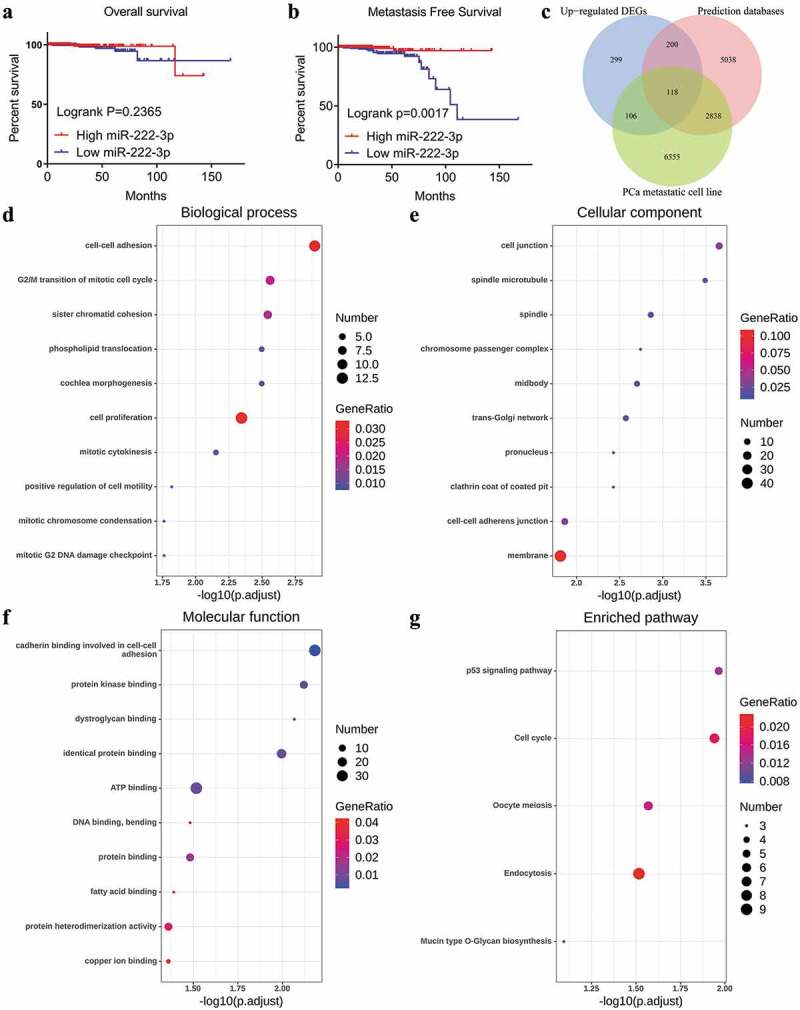
Kaplan-Meier analysis of miRNA-222-3p and the GO and KEGG pathway analyses of target genes in MPCa. (a) Overall survival of patients with different miRNA-222-3p expression in PCa. (b) Metastasis-free survival rate of patients with different miRNA-222-3p expression in PCa. (c)The Venn diagrams of potential target genes of miRNA-222-3p. (d) Biological process (BP). (e) Cellular component (CC). (f) Molecular function (MF). (g) KEGG pathway analysis. DEGs: differently expressed genes; PCa: prostate cancer; GO: Gene Ontology; KEGG: Kyoto Encyclopedia of Genes and Genomes; MPCa: metastatic prostate cancer

A total of six studies that met our study criteria were analyzed to identify the clinical significance of miRNA-222-3p in MPCa (Table 1 and Supplemental Figure S2). Based on these six studies, the expression levels of miRNA-222-3p in LPCa and MPCa samples are shown in Figure 1, and the distinguishing capacity of miRNA-222-3p in MPCa samples is shown in Figure 2. The combined standard mean difference was −0.76 (95% CI, −1.44 to −0.08), suggesting that miRNA-222-3p expression was significantly decreased in MPCa (Figure 3(a)). Sensitivity analysis showed that the outcome was stable (Figure 3(b)). Begg’s and Egger’s tests indicated that no publication bias was observed (Figure 3(c,d)). The summary receiver operating characteristic curve showed that miRNA-222-3p had a moderate potential to distinguish MPCa from LPCa (area under the curve = 0.81) (Figure 3(e)). No publication bias was observed (Figure 3(F)). Based on 27 datasets, miRNA-222-3p expression was notably decreased in PCa (Supplemental Table S1, Supplemental Figure S3 and Supplemental Figure S6). Only TCGA RNA-seq dataset was utilized to investigate the association between miRNA-222-3p expression in patients with PCa and clinical characteristics because the other datasets lacked details of the clinicopathological parameters of PCa patients. As shown in Supplemental Table S2, miRNA-222-3p was significantly decreased in PCa patients aged ≥60 years, with T3 classification, N1 classification, M1 classification, Gleason score ≥8 and disease recurrence. In addition, the Kaplan–Meier survival analysis revealed that low miRNA-222-3p expression was related to poorer metastasis-free survival (MFS) in patients with PCa, although no significant correlation was observed between miRNA-222-3p and overall survival (OS) (Figure 4(a,b)).

**Table 1.** The means and standard deviations of miRNA-222-3p expression levels in LPCa and MPCa based on six studies

				MPCa			LPCa		
Study	Country	Year	Sample type	N	M	SD	N	M	SD
GSE21036	USA	2010	tissue	14	6.770	1.443	99	8.644	0.927
GSE26964	China	2011	tissue	7	4.217	2.045	6	7.735	2.920
GSE134266	China	2019	tissue	8	2.256	0.312	20	2.211	0.112
GSE117674	Canada	2018	tissue	19	11.339	0.646	19	11.981	0.537
GSE112264	Japan	2018	tissue	63	2.454	2.470	746	2.358	2.428
TCGA	NA	NA	tissue	20	4.804	0.597	481	5.394	0.859

LPCa: localized prostate cancer; MPCa: metastatic prostate cancer; N: number; M: mean; SD: standard deviation; TCGA: The Cancer Genome Atlas.

### 3.2. Potential target genes of miRNA-222-3p in MPCa

#### 3.2.1. Gene expression data for MPCa cell lines after miRNA-222-3p transfection

Potential target genes regulated by miRNA-222-3p were acquired based on gene expression of MPCa cell lines (GSM1357604 and GSM1357687) in the GEO database. Using the criteria (|log2FC| > 1), 6,542 genes were identified in GSM1357604 and 5,732 in GSM1357687. Finally, after eliminating duplicates, we acquired 9,617 potential target genes of miRNA-222-3p in MPCa.

#### 3.2.2. Upregulated genes among the DEGs in MPCa

Based on 10 studies of MPCa, genes that appeared at least three times among the groups of upregulated DEGs. As a result, 723 genes were deemed to be the potential target genes of miRNA-222-3p.

#### 3.2.3. Online prediction databases

In total, 8,194 potential target genes of miRNA-222-3p were selected via miRWalk2.0. By intersecting the genes that were selected from the three different methods described in Section 2.2, we identified 118 potential target genes of miRNA-222-3p for further investigation (Figure 4(c)).

### 3.3. GO, KEGG and PPI network analyses

The GO enrichment analysis was performed to determine the functional annotations of 118 potential target genes of miRNA-222-3p. The top 10 GO functional annotations for the potential target genes of miRNA-222-3p in the biological process (BP), cellular component (CC) and molecular function (MF) categories are presented in Figure 4(d–f), respectively. The most enriched GO terms were ‘cell-cell adhesion’ in BP, ‘cell junction’ in CC, and ‘cadherin binding involved in cell-cell adhesion’ in MF. According to the KEGG pathway analysis, the most remarkable enrichment of potential target genes of miRNA-222-3p was in the ‘p53 signaling pathway’ (Figure 4(g)). In the PPI network analysis, SNAP91 showed the highest connectivity among the hub genes and was selected for subsequent analysis (Supplemental Figure S4).

### 3.4. The correlation between miRNA-222-3p and SNAP91

**Figure 5.**
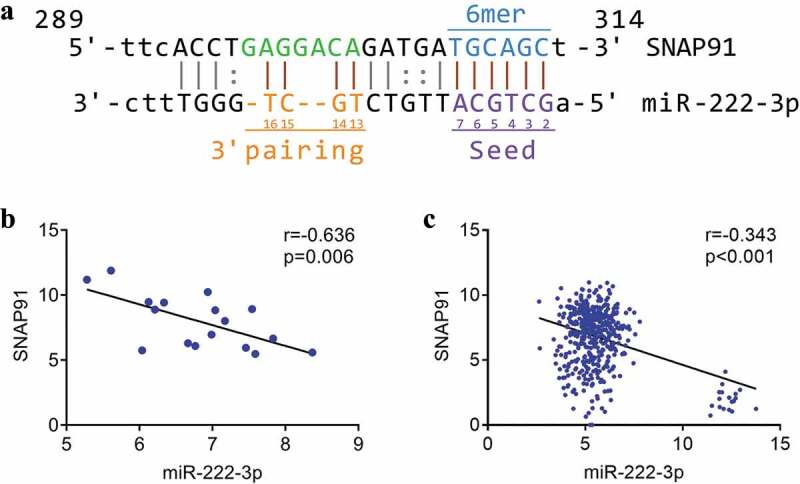
The association between miRNA-222-3p and SNAP91. (a) The binding sequence between SNAP91 and miRNA-222-3p. (b) Spearman’s correlation analysis between miRNA-222-3p and SNAP91 in MPCa. (c) Spearman’s correlation analysis between miRNA-222-3p and SNAP91 in PCa. SNAP91: synaptosome-associated protein 91; MPCa: metastatic prostate cancer; PCa: prostate cancer

We identified complementary binding sequences consisting of seven base-pairs between miRNA-222-3p and SNAP91 (Figure 5(a)). Spearman’s correlation analysis revealed a significant inverse correlation between miRNA-222-3p expression and SNAP91 expression both in MPCa (r = −0.636, P = 0.006) and PCa (r = −0.343, P < 0.001) (Figure 5(b,c)). Furthermore, we found that the expression of miRNA-222-3p was negatively correlated with SNAP91 expression in 14 different cancers (Supplemental Figure S5).

### 3.5. Clinical significance of SNAP91 in MPCa

**Figure 6.**
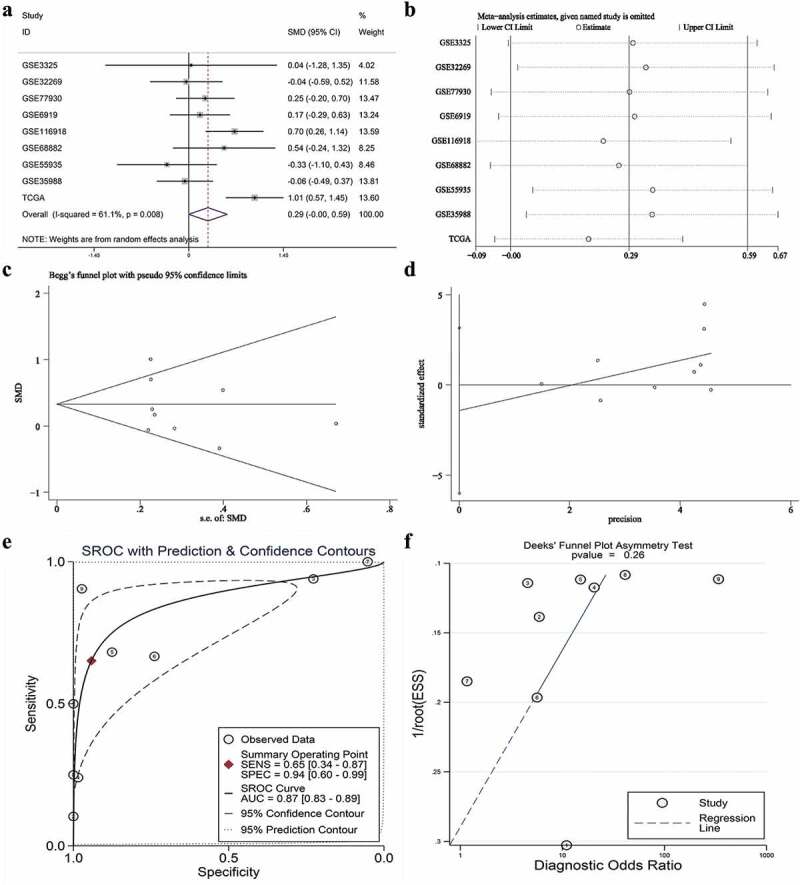
The expression and distinguishing capacity of SNAP91 in MPCa. (a) Forest plot. (b) Sensitivity analysis plot. (c) The Begg’s funnel plot of the publication bias. (d) Egger’s publication bias plot. (e)SROC curve. (f) Deek’s funnel plot test. TCGA: The Cancer Genome Atlas; standard mean difference: standard mean difference; CI: confidence interval; area under the curve: area under the curve; SROC: summarized receiver operating characteristic; SNAP91: synaptosome-associated protein 91; MPCa: metastatic prostate cancer

**Figure 7.**
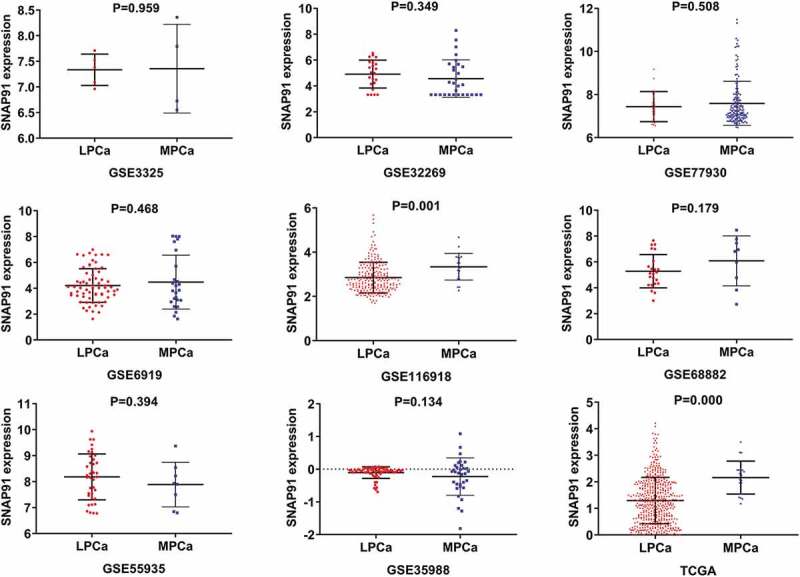
Expression levels of SNAP91 between LPCa samples and MPCa samples. TCGA: The Cancer Genome Atlas; SNAP91: synaptosome-associated protein 91; LPCa: localized prostate cancer; MPCa: metastatic prostate cancer

**Figure 8.**
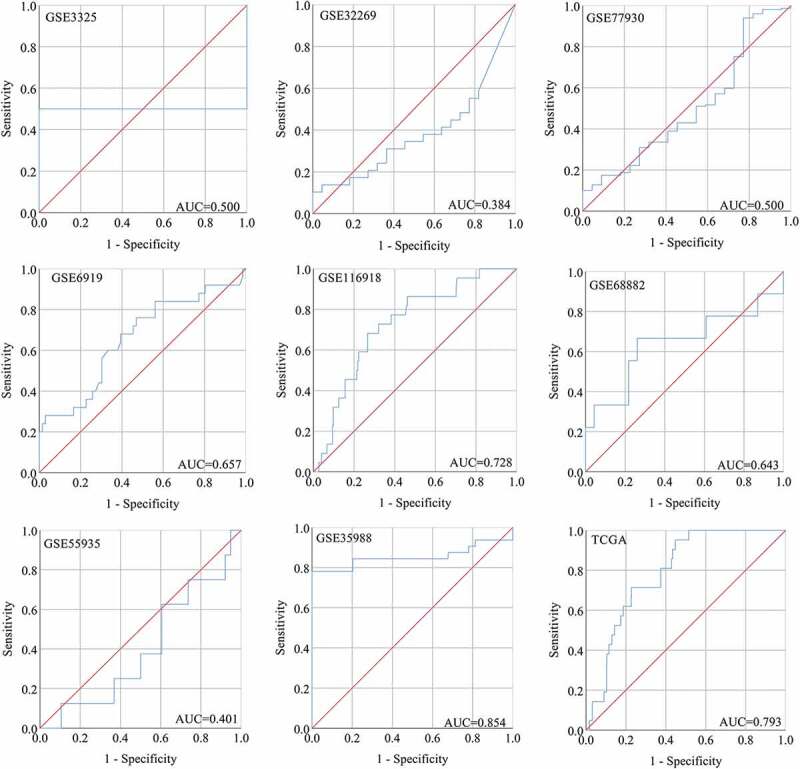
The ROC curves of SNAP91 for distinguishing capacity in MPCa samples. area under the curve: area under the curve; TCGA: The Cancer Genome Atlas; ROC: receiving operator characteristic; SNAP91: synaptosome-associated protein 91; MPCa: metastatic prostate cancer

**Figure 9.**
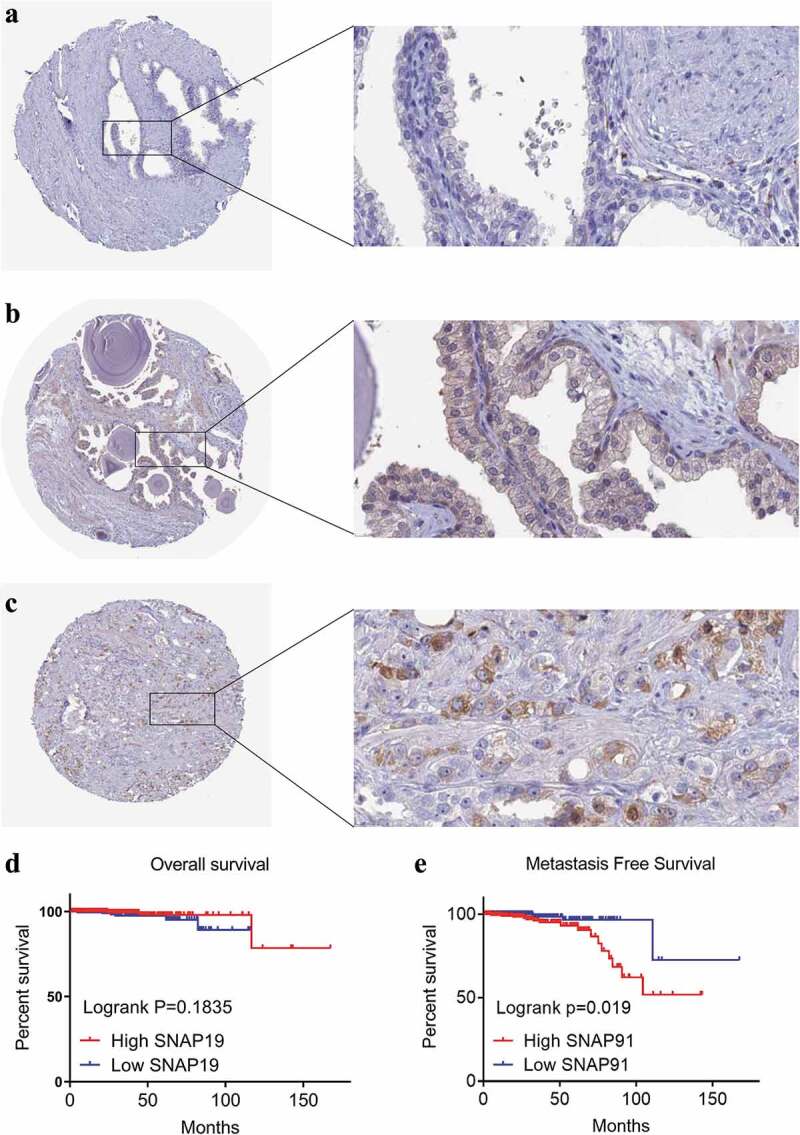
HPA immunohistochemistry and Kaplan-Meier analysis of SNAP91. (a) The expression level of SNAP91 protein was not detected in normal prostate tissue (Antibody HPA029633, Male, age 60). (b) The expression level of SNAP91 protein was not detected in normal prostate tissue (Antibody HPA029632, Male, Age 48). (c) The expression level of SNAP91 protein was moderate in prostate adenocarcinoma (Antibody HPA029633, Male, age 79). (d) Overall survival of patients with different SNAP91 expression in PCa. (e) Metastasis-free survival rate of patients with different SNAP91 expression in PCa. HPA: Human Protein Atlas; SNAP91: synaptosome-associated protein 91

After screening, we identified nine studies containing SNAP91 expression data in MPCa (Table 2 and Supplemental Figure S7). Based on these nine studies, the differential expression of SNAP91 in LPCa and MPCa samples is shown in Figure 7, and the distinguishing ability of SNAP91 in MPCa samples is shown in Figure 8. Random-effect models were used to merge the standard mean difference due to the heterogeneity in the data among individual studies (I^2^ = 61.1%). According to the combined results, SNAP91 was upregulated in 299 MPCa samples compared to 938 LPCa samples (Figure 6(a–d)). In addition, the summary receiver operating characteristic curves showed that the area under the curve was 0.87 (95% CI: 0.83 to 0.89) for SNAP91 in distinguishing between MPCa and LPCa (Figure 6(e,f)). These results indicated that SNAP91 was upregulated in MPCa and had a moderate ability to differentiate MPCa from LPCa. SNAP91 was found to be notably upregulated in PCa based on analysis of 16 datasets with 1,176 PCa samples and 495 non-PCa samples (Supplemental Table S3, Supplemental Figure S8 and Supplemental Figure S9). Based on the HPA database, the SNAP91 protein expression level was moderate in PCa samples, while SNAP91 protein expression was not detected in normal samples (Figure 9(a–c)). Evaluation of the clinical significance of SNAP91 in PCa in TCGA database suggested that SNAP91 was significantly upregulated in PCa patients with M1 classification, Gleason score ≥8 and disease recurrence (Supplemental Table S4). The Kaplan–Meier survival analysis revealed that patients with higher SNAP91 expression had a shorter MFS, while there was no significant correlation between SNAP91 expression and OS (Figure 9(d,e)).

**Table 2.** The means and standard deviations of SNAP91 expression levels in LPCa and MPCa based on nine studies

				MPCa			LPCa		
Study	Country	Year	Sample type	N	M	SD	N	M	SD
GSE3325	USA	2005	tissue	4	7.356	0.865	5	7.334	0.306
GSE32269	USA	2011	tissue	29	4.565	1.454	22	4.914	1.079
GSE77930	USA	2016	tissue	149	7.589	1.022	22	7.440	0.700
GSE6919	USA	2007	tissue	25	4.476	2.089	66	4.210	1.301
GSE116918	UK	2018	tissue	22	3.339	0.746	225	2.850	0.692
GSE68882	USA	2015	tissue	9	6.084	1.928	23	5.280	1.288
GSE55935	Norway	2014	tissue	8	7.886	0.859	38	8.181	0.885
GSE35988	USA	2012	tissue	32	−0.227	0.573	59	−0.104	0.177
TCGA	NA	NA	tissue	21	2.163	0.432	478	1.295	0.873

SNAP91: synaptosome-associated protein 91; LPCa: localized prostate cancer; MPCa: metastatic prostate cancer; N: number; M: mean; SD: standard deviation; TCGA: The Cancer Genome Atlas.

## 4. Discussion

In this study, we investigated the clinical significance of miRNA-222-3p and its potential molecular mechanism in MPCa. Our analysis of 1,502 samples from miRNA chip and miRNA sequencing datasets revealed downregulated expression of miRNA-222-3p in MPCa. SNAP91 was identified as a hub gene in MPCa, and a significant negative correlation was identified between miRNA-222-3p and SNAP91 in MPCa. Our analysis of 1,237 samples showed that SNAP91 was highly expressed in MPCa. In addition, the downregulation of miRNA-222-3p and upregulation of SNAP91 have moderate potential for distinguishing MPCa from LPCa and are associated with worse metastasis-free survival in MPCa. Our results indicated that miRNA-222-3p might play an important role in MPCa by regulating SNAP91 expression.

The expression of miRNA-222-3p in PCa has been widely reported, although the results are controversial. In some studies, miRNA-222-3p was found to be highly expressed in PCa tissues and functioned as a proto-oncogene [26–29]. However, other studies showed that miRNA-222-3p was downregulated in PCa tissues and was considered to function as a tumor suppressor factor [30–32]. Based on 1,480 PCa tissue samples, we found that miRNA-222-3p expression was lower in PCa than that in non-PCa, and the large sample sizes lend credibility to these results. Reports of the expression of miRNA-222-3p in body fluids in PCa patients are inconsistent. Jacob et al. found that miRNA-222-3p was significantly downregulated in 215 urine samples from PCa patients compared with the levels detected in 29 urine samples from non-PCa individuals [28]. In a study of plasma samples from 70 patients and urine samples from 33 patients before radical prostatectomy, Nikhil et al. found high miRNA-222-3p expression in urine, while there was no significant change in the levels detected in plasma samples [29]. In our study, we found that there was no significant difference in the expression of miRNA-222-3p between 225 PCa samples and 204 control samples, which may be related to the small sample size and the source of body fluids (including serum, plasma and urine). Therefore, the expression of miRNA-222-3p in the body fluids of PCa patients requires further investigation in a larger sample.

To the best of our knowledge, there are only two reports of the expression of miRNA-222-3p in MPCa. Mercatelli et al. found that miRNA-222-3p was highly expressed in metastatic bone tumors [25]. In another study, miR-222 was shown to be downregulated in MPCa [15]. These contradictory results of these studies prompted us to verify the expression of miRNA-222-3p in MPCa through analysis of a large sample. In this study, we analyzed 1,502 samples and found that miRNA-222-3p was significantly downregulated in MPCa. We also found that the downregulated expression of miRNA-222-3p had a moderate ability to distinguish MPCa from LPCa (area under the curve = 0.81). By analyzing the PCa samples in TCGA database, we observed that the low miRNA-222-3p expression was associated with worse clinicopathological results in PCa patients, although there was no obvious correlation with OS, which may be related to the relatively high five-year OS rate in PCa patients. In addition, we observed that patients with low miRNA-222-3p expression have worse MFS. Thus, our results implicate miRNA-222-3p as a biomarker of MPCa.

We screened 118 potential target genes of miRNA-222-3p using three methods. KEGG pathway analysis showed that the potential target genes of miRNA-222-3p were significantly enriched in the p53 signaling pathway. Previous studies have indicated that the p53 signal pathway is involved in the process of tumor metastasis. For example, activation of the p53 pathway inhibits metastasis in colorectal cancer [33] and the activation of the p53 pathway weakened metastasis and invasion in gastric cancer [34]. Furthermore, Yang et al. found that p53 pathway may be regulated by SEMA4C, which has an impact on the metastasis and progression of breast cancer [35]. In addition, p53 can mutate into p53S to induce the growth and metastasis of PCa cells [36]. Therefore, we speculate that miRNA-222-3p participates in PCa metastasis by regulating the p53 signal pathway, although this hypothesis requires verification in further studies.

In the PPI network analysis, SNAP91 had the highest degree of connectivity and was identified as a hub gene. Using the miRWalk2.0 database, we identified complementary binding sequences consisting of seven base-pairs between miRNA-222-3p and SNAP91. By analyzing TCGA MPCa samples, we found a significant negative correlation between miRNA-222-3p and SNAP91. Therefore, we speculated that the negative regulation of SNAP91 by miRNA-222-3p was involved in the metastasis of PCa. SNAP91 encodes clathrin coat assembly protein 180 (AP180) and is critical for the synthesis and effect of clathrin-coated vesicles, which are the main way of recovering vesicles of the presynaptic membrane [37]. SNAP91 is mainly distributed in the polar part of the synapse, participating in the vesicular transport of neurotransmitters [38]. Previous studies mainly reported the relationship between SNAP91 and nervous system diseases. Yemni et al. identified SNAP91 as one of the susceptibility genes of Parkinson’s disease through the method of whole exome sequencing, and involuntary movement was observed in the SNAP91 transgenic mice [39]. AP180 knockout mice showed excitatory/inhibitory imbalance, which underlies epilepsy [40]. SNAP91 was reported as one of the susceptibility genes of schizophrenia [41,42]. SNAP91 protein level in patients with Alzheimer’s disease was significantly decreased, and the reduction of AP180 resulted in synaptic dysfunction, which might be associated with cognitive deficit [43]. At present, only few studies reported SNAP91 in cancer. Pan et al. found that miRNA-301b promoted the development of human esophageal cancer by targeting SNAP91, and the downregulation of SNAP91 in esophageal cancer tissues was related to the poor prognosis of patients [44]. Gao et al. reported that SNAP91 was reduced in glioblastoma and conversely associated with glioma grade [45]. Thus far, no study has mentioned SNAP91 in PCa. In this study, our analysis of the data from 1,671 samples showed that SNAP91 mRNA expression was upregulated in PCa. Immunohistochemistry results showed that SNAP91 protein expression was also highly expressed in PCa. Our analysis of 1,237 samples revealed that SNAP91 expression was increased in MPCa (standard mean difference = 0.29), and had a moderate ability to discriminate between MPCa and LPCa (area under the curve = 0.87). TCGA database analysis showed that high SNAP91 expression was associated with poor clinicopathological outcomes and worse MFS. Our results show that SNAP91 is associated with the metastatic phenotype of PCa and may facilitate tumor metastasis in PCa, which needs verification in our continued studies.

Although our study provides some new clues to the molecular mechanism of MPCa, some limitations should be noted. First of all, there was a significant heterogeneity in the data analyzed in our study, which reduces the credibility of our results to a certain extent. Second, the expression and diagnostic potential of miRNA-222-3p and SNAP91 were studied based on tissue samples, and their diagnostic value in MPCa requires further investigation based on body fluid samples. Third, although we evaluated the clinical significance of miRNA-222-3p and SNAP91 in MPCa, their biological functions and relationships in MPCa require further investigation both in vivo and in vitro.

## 5. Conclusion

In summary, our results suggest that miRNA-222-3p is significantly downregulated in MPCa (standard mean difference = −0.76, area under the curve = 0.81), and it may participate in the metastasis of PCa by negatively regulating the expression of SNAP91. MiRNA-222-3p might be a potential biomarker and therapeutic target of MPCa.

## Supplementary Material

Supplemental Material

## Highlights

1. Low miRNA-222-3p expression was detected in metastatic prostate cancer (MPCa) (standard mean difference = −0.76).
2. SNAP91 was identified as one of hub genes of miRNA-222-3p in MPCa.
3. MiRNA-222-3p was negatively correlated with SNAP91 in MPCa (r = −0.636, P = 0.006).
4. High SNAP91 expression was detected in MPCa (standard mean difference = 0.29).
5. MiRNA-222-3p could discriminate MPCa from LPCa (area under the curve = 0.81).
