# Supplementary figures and images for: Clinical significance and potential molecular mechanism of miRNA-222-3p in metastatic prostate cancer
Source: Bioengineered. 2021 Jan 12;12(1):325–40. doi: 10.1080/21655979.2020.1867405 (PMC8806336; doi:10.1080/21655979.2020.1867405)

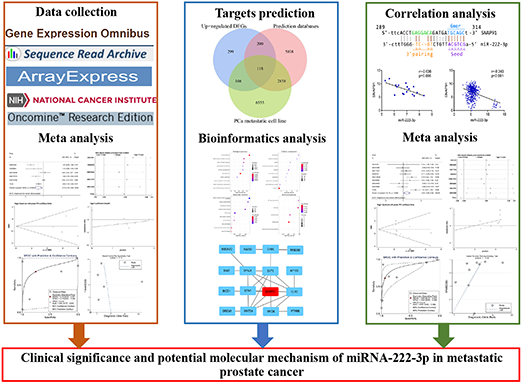

Supplement: Supplemental Material [file KBIE_A_1867405_SM4652.tif]
